# A controlled clinical trial investigating the effects of stretching and compression exercises on electromyography of calf muscles in chronic LBP patients with a deep gluteal syndrome

**DOI:** 10.1186/s13102-023-00802-4

**Published:** 2024-01-10

**Authors:** MohammadBagher Shamsi, Maryam Mirzaei, Kevork Hopayian

**Affiliations:** 1 Department of Physiotherapy, School of Rehabilitation Sciences, Kermanshah University of Medical Sciences, Kermanshah, Iran; 2 Centre for Primary Care and Population Health, University of Nicosia Medical School, Nicosia, Cyprus

**Keywords:** Deep gluteal syndrome, Electromyography, Flexion-relaxation ratio, Muscle stretching exercises

## Abstract

**Background:**

In deep gluteal syndrome (DGS), the piriformis muscle could impinge the sciatic nerve. The FAIR (flexion adduction internal rotation) test is a provocation test used to identify sciatic nerve irritation caused by this muscle. Compression and stretching exercises are usually prescribed to treat this syndrome. The aim of this study was to compare the effects of these two treatments on surface electromyography (sEMG) of the gastrocnemius and tibialis anterior in patients with low back pain (LBP) and DGS.

**Materials and methods:**

Forty-five participants were allocated to three groups of stretching exercise, compression or control. In addition to 15 min of heat and 15 min of electrical nerve stimulation for pain relief, participants in the compression exercise (CE) group received self-compression exercise, those in the stretching exercise (SE) group received self-stretching exercise and those in the control group received no extra interventions. For the two intervention groups, three sets of two minutes of exercise with two minutes of rest in between were applied. The sEMG amplitude values of the gastrocnemius and tibialis anterior muscles of the affected buttock side of any one group while performing the FAIR test were compared to the others. Pain and disability were assessed and the changes were compared between the two groups.

**Results:**

After the intervention period, no group demonstrated a change in the sEMG of the gastrocnemius or tibialis anterior muscles (*p* > 0.05). There was no difference in the change in this variable between groups (Mean difference (95% CI) of gastrocnemius was ranged over= -4.04 to 7.72 (-19.44 to 23.14); *p* = 0.603); (Mean difference (95% CI) of tibialis anterior muscles was ranged from − 2.44 to -6.43 (-18.28 to 9.31); *p* = 0.550).; Pain and disability also decreased significantly in all three study groups (*p* < 0.05). However, only the disability of patients who performed stretching exercises improved compared to the compression exercise group (Mean difference (95% CI) = -12.62 (-20.41 to -4.38); *p* = 0.009).

**Conclusion:**

Neither stretching nor compression exercises altered the sEMG of the gastrocnemius and tibialis anterior muscles in patients with DGS. Furthermore, performing stretching exercises improved disability compared to the other interventions.

**Trial registration:**

The trial was retrospectively registered in the Iranian Registry of Clinical Trials (www.irct.ir) on 10/01/2017 as IRCT201604178035N4. URL of the record: https://en.irct.ir/trial/8473.

## Background

Low back pain (LBP) is determined as pain between the lower border of the twelfth rib and the lower gluteal folds, usually associated with painful limitation of movement [1]. LBP and sciatica are among the top ten causes of years lived with disability in developing countries and the leading cause in 45 developed nations [2]. The incidence of sciatica in Western countries is estimated to be 5 per 1000 [3]. One of the most common conditions associated with LBP is sciatica, with lifetime prevalence ranging from 1.2 to 43% [4]. Many musculoskeletal problems may have compression on the sciatic nerve as it crosses the deep gluteal space [5]. This disorder is called deep gluteal syndrome (DGS). In the past, it was known as piriformis syndrome (PS) because the piriformis muscle was the first to be affected [5]. The sciatic nerve can be impinged by the piriformis muscle, causing pain in the buttocks, sciatica or both [5]. The FAIR (flexion adduction internal rotation) test is a provocation test used to detect irritation of the sciatic nerve caused by the piriformis muscle. While the patient is lying supine with the knee flexed 90 degrees, the hip on the affected side is passively placed in 90 degrees of flexion, horizontal adduction, and internal rotation [6]. The movement of the hip joint into flexion, adduction, and internal rotation causes stretching of the tight piriformis muscle and, theoretically, compresses the sciatic nerve and causes tenderness and pain. In the field of peripheral nerve pathologies, the concept of “dynamic entrapment” has recently been introduced. In general, adduction, internal rotation, and flexion (FAIR position) of an affected leg, cause aggravation of the painful symptoms of PS. Using dynamic MRI imaging, it is shown that in the FAIR position, the infra-piriformis foramen narrows and the sciatic nerve is closer to the ischial spine, and the piriformis muscle becomes tighter against the sciatic nerve and causes lateralization and anterior excursion of the nerve and also causes a transient reduction in the nerve conduction velocity at that point [7, 8].

Conservative treatments encompass a range of therapeutic approaches, including manual therapies, local injections of various substances like corticosteroids, and acupuncture [9]. Among these, compression techniques and (self) stretching exercises are frequently recommended by therapists due to their ease of application.

Various outcome measures have been employed to assess the effectiveness of back pain therapies, such as visual and numerical analogue scales of pain and disability. In this study a different approach, surface electromyography (sEMG) is taken. Electromyography is a well-established method of recording the electrical activity produced by skeletal muscle contraction. More recently, surface EMG has gained prominence as a convenient non-invasive alternative convenient alternative using surface electrodes obviating the need for needle insertion into muscles. Nowadays, sEMG applications extend to diverse fields, including exercise and sports pathophysiology, movement analysis, ergonomics and occupational medicine, and in a number of related fields [10]. Research in this area is crucial as it can shed light on the potential effectiveness of interventions, ultimately leading to improvements in managing disorders like deep gluteal syndrome (DGS) that affect a significant number of patients. We hypothesized that the impact of interventions on DGS and the reduction of sciatic nerve compression would reflect in the sEMG of leg muscles innervated by this nerve. In our study, we aimed to compare the effects of compression and stretching exercises on the sEMG of the gastrocnemius and tibialis anterior muscles, both of which are innervated by the sciatic nerve. Given the high prevalence of DGS, particularly in societies with demanding physical work, any improvements in related interventions could have a direct and positive impact on physical health and well-being.

## Methods

This was a single-blind, randomized clinical trial with three parallel groups, conducted in a university clinic located in Kermanshah, Iran, following the CONSORT guidelines. The trial protocol received approval from the Ethics Committee of Kermanshah University of Medical Sciences (Approval ID: KUMS.rec.1395.169) and was registered on the Iranian registry of the clinical trial website (www.irct.ir) on 10/01/2017, with the identification number IRCT201604178035N4.

### Participants

The positive stretch provocation test of FAIR, combined with tenderness over the piriformis muscle upon palpation, as determined by an expert physiotherapist, served as the basis for diagnosing DGS during the physical examination. Participants eligible for inclusion in the study were individuals aged 18–60 who experienced lower back pain (LBP) within the area between the lower rib cage and gluteal folds. Exclusion criteria were applied to those with other forms of LBP, such as disc herniation or spondylolisthesis, as well as individuals with hip pathology, recent hip or knee injuries, or systemic diseases impacting their overall health, such as severe diabetes and obesity The exclusion process involved a combination of medical history assessment, radiologic imaging, and clinical tests designed to rule out other potential causes of LBP, such as intervertebral disc herniation or spondylolisthesis.

A total of 45 participants (32 females and 13 males) were included in the study. The mean age (SD) of the participants was 41.80 (9.59) years, and the mean (SD) body mass index was 26.48 (3.21). All participants provided written, informed consent to take part in the trial.

Randomization sequences were generated by a statistician who was not involved in the clinical procedures. A randomized block procedure with a block size of 3 was used. All enrolled participants were randomly assigned to either a control group or a treatment group. The principal assessor (MB.Sh) responsible for clinical data collection was kept blind to the assignment of patients.

The sample size was determined using the Pocock formula, employing G-Power software and drawing on primary outcome data from a prior study [11] (m1 = 3.36, SD1 = 1.58, m2 = 3.36, SD2 = 2.06). With a 95% confidence level and 80% power, it was calculated that 15 participants were required for both the control and treatment groups.

### Interventions

All participants received a standardized treatment protocol consisting of 15 min of heat therapy (Hot Pack) and 15 min of transcutaneous electrical nerve stimulation (TENS) applied to the low back and buttock area. In addition, the intervention groups received more specific treatments [9].

#### Compression exercise

**Fig. 1:**
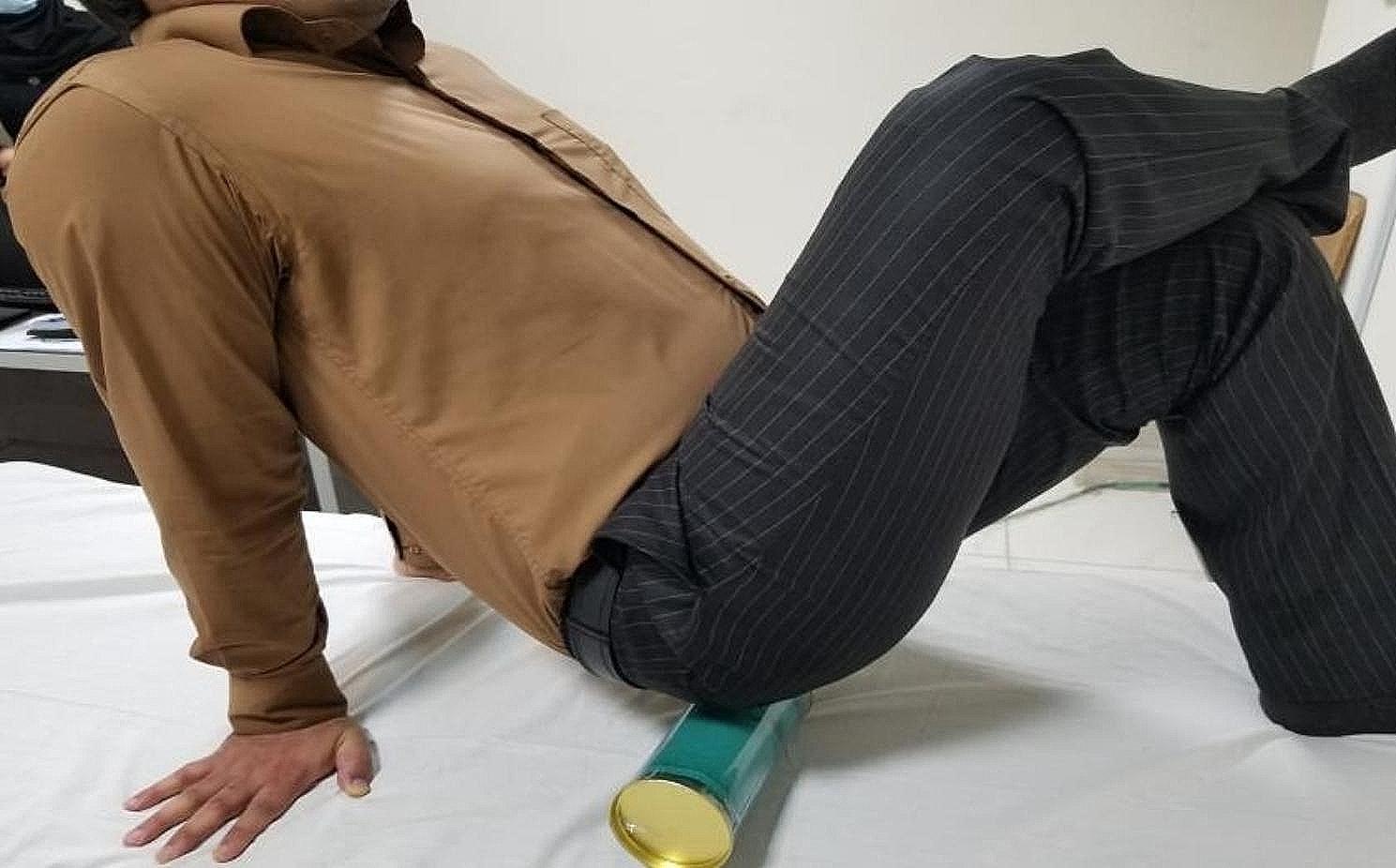
Compression exercise

Participants in the compression exercise (CE) group assumed a lying position with the ankle of the tested side resting on the other flexed knee to stretch the muscle. A foam roller was placed under the hip, and they gently rolled on the lateral buttock area at the point of tenderness (see Fig. 1).

**Fig. 2:**
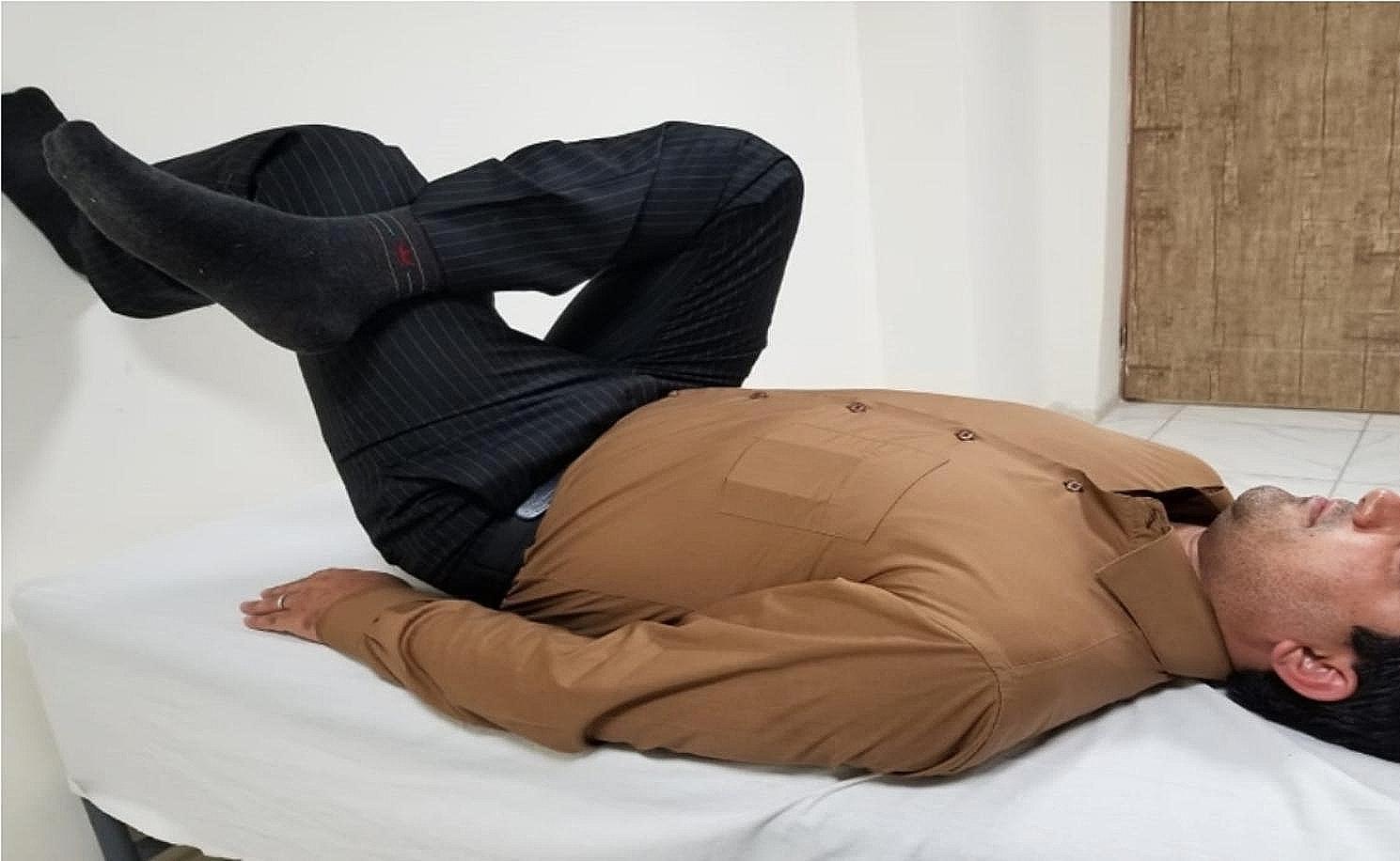
Stretching exercise

Stretching exercise (SE): Participants in the stretching exercise (SE) group were positioned supine with their arms at their sides and palms facing downward. Their healthy foot was placed against a wall and maintained in that position. While lying on their back, they placed the ankle of the affected side over the other knee, just above the kneecap, with the leg bent. Participants were asked if they felt a slight stretch in the piriformis muscle (Fig. 2). Both intervention groups performed three sets of two-minute exercises with two minutes of rest between each set. The control group did not receive any additional interventions.

To ensure that the exercises were performed correctly, an experienced physiotherapist closely monitored the participants. Each group received three sessions of physiotherapy treatment per week, totaling ten sessions.

### Outcome measures

#### Primary outcome

The primary outcome measures the difference in sEMG amplitude values between the gastrocnemius and tibialis anterior muscles on the affected buttock side within each group when compared to the other groups.

#### Secondary outcome

The secondary outcome evaluates differences in pain or disability among the groups, comparing each group to the others.

To gain insights into the pressure on the sciatic nerve, we assessed the electromyographic activity of muscles innervated by the sciatic nerve, specifically the gastrocnemius and tibialis anterior muscles. This assessment was conducted during the “Flexion Adduction and Internal Rotation test (FAIR),” which allowed us to acquire EMG signals from these muscles while stretching the piriformis muscle.

### Surface Electromyography (sEMG)

EMG signals were recorded using the Myon 320 device (Myon AG, Switzerland) with pre-gelled self-adhesive surface electrodes made of Ag/AgCl. To reduce skin impedance, the skin was abraded and cleaned with alcohol wipes. Electrode locations were determined following the SENIAM guideline (http://www.seniam.org/).

The placement for each muscle was as follows:

Gastrocnemius: Positioned on the most prominent bulge of the medial muscle.

The equipment had a common mode rejection ratio (CMRR) of 110 dB and a sampling rate of 1,000 Hz. A bandpass filter was applied, with a range between 20 and 450 Hz.

Tibialis Anterior: Placed at a location one-third of the distance between the tip of the fibula and the tip of the medial malleolus. The inter-electrode distance was 2 cm.

The equipment had a common mode rejection ratio (CMRR) of 110 dB and a sampling rate of 1,000 Hz. A band pass filter was applied, with a range between 20 and 450 Hz. sEMG signals from the gastrocnemius and tibialis anterior muscles were recorded while participants performed the Flexion Adduction and Internal Rotation (FAIR) test. Additionally, sEMG of these muscles was recorded while participants made maximal efforts to plantar and dorsiflex their ankle in the supine position, respectively. The root mean squares (RMS) of these sEMG signals were calculated. To normalize the sEMG of muscles during the FAIR test, the RMS of each muscle was divided by the RMS of the Maximum Voluntary Contraction (MVC) signal of the same muscle. The normalized RMS values were used as the outcome measure for assessment.

The pain intensity of participants was measured using the visual analogue scale (VAS), where 0 indicated no pain, and 100 represented pain as severe as possible. Disability was assessed using the Persian-translated version of the Oswestry Disability Questionnaire, with 0 indicating no disability and 100 representing total disability [12].

### Statistical analysis

Sample characteristics were presented as mean (SD) and counts (percentage) for continuous, and categorical variables, respectively. One-way ANOVA and Fisher’s exact tests were applied to sample characteristics comparison.

2 times (pre vs. post) × 3 groups (two different types of exercises vs. control) repeated-measures ANOVA was conducted on all outcome measures (EMG signals, pain and disability) to examine the main effects of the time and interaction. The main effect of the group (as a between-subjects factor) was determined by any conspicuous difference in outcome measures observed between the three groups. So, analysis of covariance (ANCOVA) with LSD post hoc test was performed on the outcome measures to explore the between-group differences. Partial eta-squared was used for effect size.

All statistical analyses were performed using SPSS software version 26 and *p* < 0.05 were considered the significant level.

## Results

**Fig. 3:**
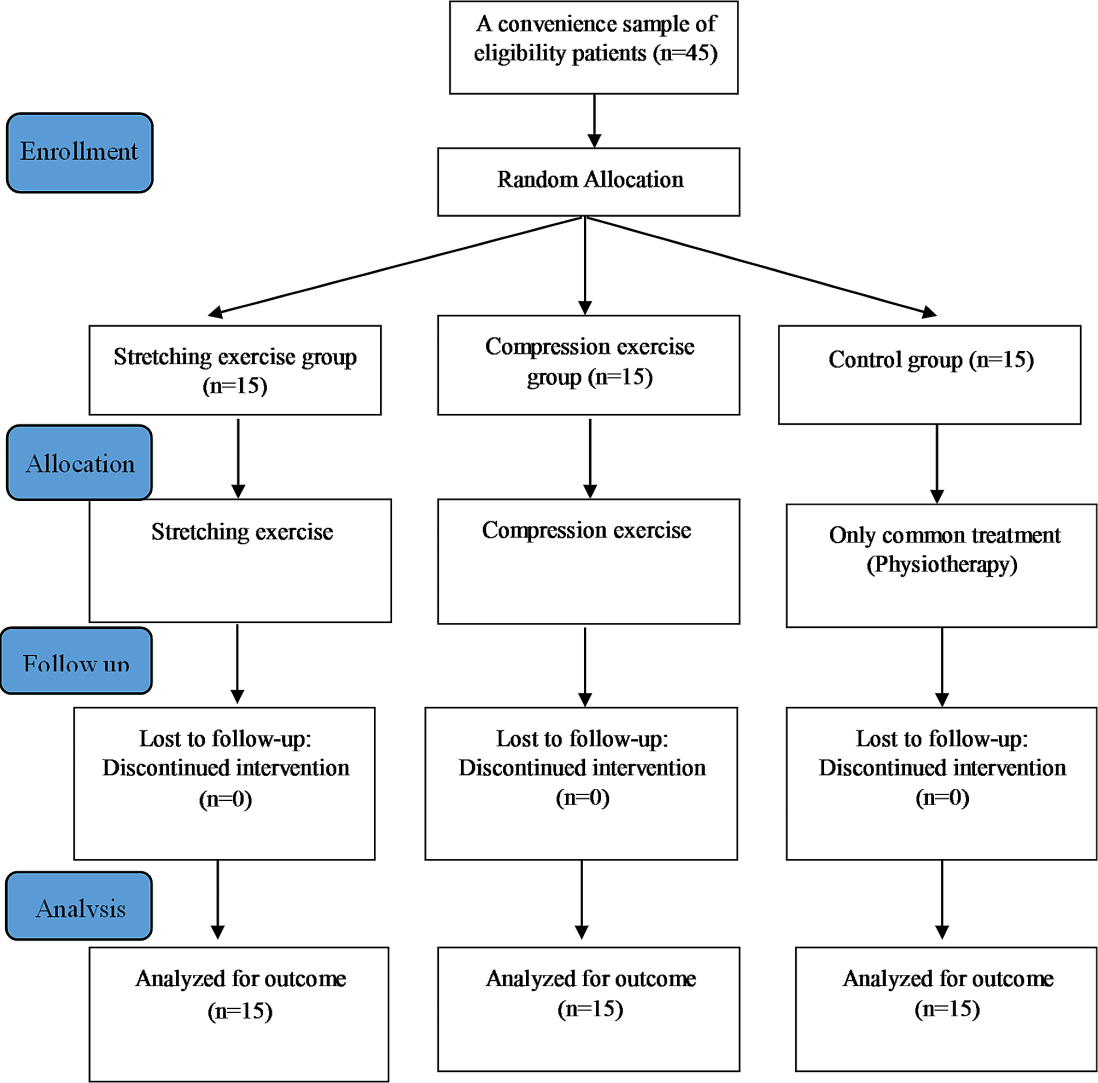
Flow of study design

The study started with 45 participants, and there was no loss to follow-up, meaning that all participants who were assigned to the groups received the allocated intervention throughout the study (Fig. 3). The demographic characteristics of the three groups were compared, and there were no statistically significant differences between the groups (all *p*-values > 0.05). This suggests that the groups were comparable in terms of these characteristics. (Table 1).

**Table 1:** Comparison of baseline demographic and background characteristics between groups

Characteristics	Control (*n* = 15)	Compression Exercise (*n* = 15)	Stretching Exercise (*n* = 15)	*p*-value
Age(year)	42.07 (9.94)	39.67 (9.69)	43.67(9.37)	0.527^#^
Height (cm)	165.00(8.87)	163.33(6.23)	167.47(7.28)	0.329^#^
Weight (kg)	69.20 (8.81)	70.40 (10.97)	77.33 (8.26)	0.07^#^
BMI (kg/m^2^)	25.49(3.07)	26.34(3.46)	27.62(2.93)	0.192^#^
Sex				
Female	11 (73.3)	13 (86.7)	8 (53.3)	0.320*
Male	4 (26.7)	2 (13.3)	7 (46.7)	

BMI: Body Mass Index/ Data are means (SD) except sex that presented as number (percent)/ ^**#**^Based on one-way ANOVA test/ ^*^Based on Fisher’s exact tests

### Primary outcome

**Fig. 4:**
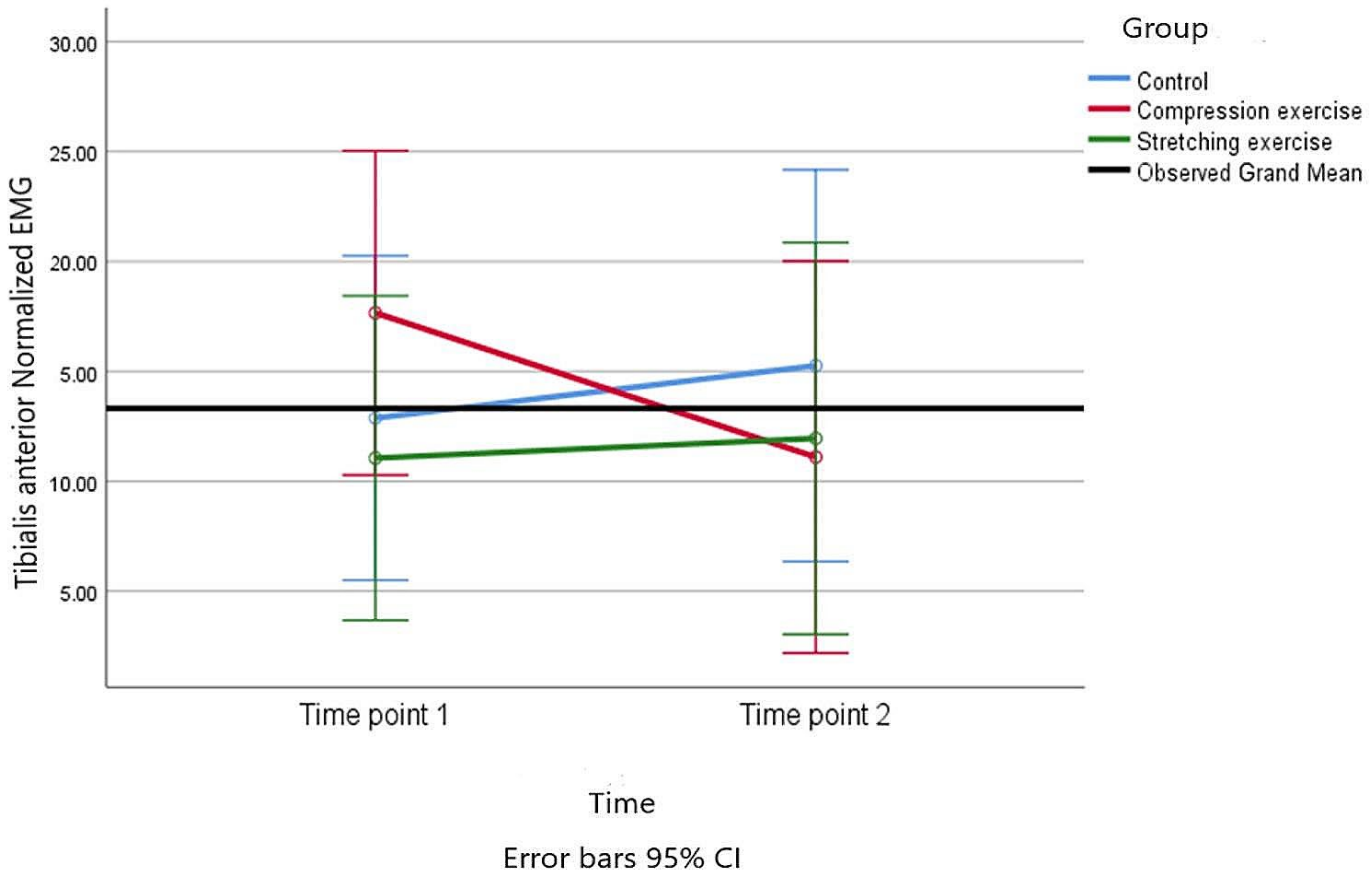
Trends of the Tibialis anterior normalized EMG for the three groups at the two time points of the study

**Fig. 5:**
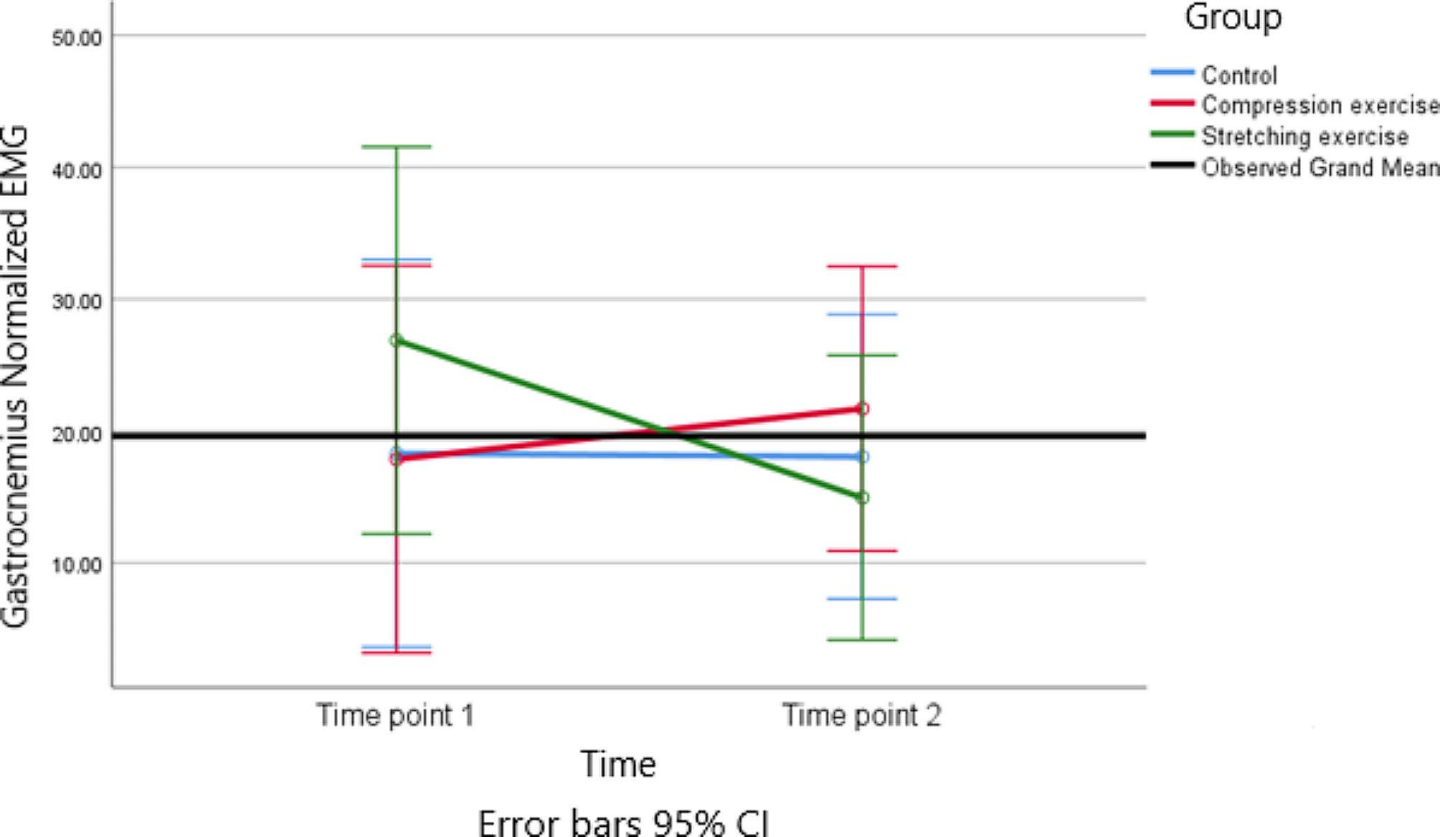
Trends of the Gastrocnemius normalized EMG for the three groups at the two time points of the study

) A repeated-measures ANOVA was conducted to analyze the EMG signals. This analysis involved comparing measurements taken at two different times (pre vs. post) across the three intervention groups. The results showed that none of the main effects or interaction effects were statistically significant (*p* > 0.05). The effect sizes ranged from 0.004 to 0.51. (Table 2) (Figs. 4 and 5). In term of gastrocnemius muscles, pairwise comparison showed that the mean difference (95% CI) of CE vs. control group, CE vs. SE and SE vs. control group were 3.68 (-11.59 to 18.97), 7.72 (-7.69 to 23.14), and − 4.04 (-19.44 to 11.37), respectively.

**Table 2:** Comparison of EMG signals (normalized RMS) among the three groups of study (*n* = 45)

Variables	Measurement period	Before Intervention	After Intervention	Main effect of group	Main effect of time	Interaction effect (time*group)
Gast	Compression Exercise	17.86 (11.78)	21.69 (23.45)	p = 0.603; F = 0.512; df = 2; effect size = 0.024	p = 0.567; F = 0.332; df = 1; effect size = 0.008	p = 0.393; F = 0.956; df = 2; effect size = 0.044
	Stretching Exercise	26.87 (42.26)	14.94 (13.72)			
	Control	18.31 (21.33)	18.1 (23.39)			
TibAnt	Compression Exercise	17.66 (18.16)	11.09 (12.10)	p = 0.550; F = 0.607; df = 2;	p = 0.676; F = 0.178; df = 1; effect size = 0.004	p = 0.331; F = 1.13; df = 2; effect size = 0.051
	Stretching Exercise	11.05 (10.89)	11.94 (21.18)	effect size = 0.029		
	Control	12.87 (12.37)	15.25 (16.75)			

Gast (Gastrocnemius), TibAnt (Tibialis Anterior), Mean (SD) and Effect size were reported,

In term of tibialis anterior muscles, pairwise comparison showed that the mean difference (95% CI) of CE vs. control group, CE vs. SE and SE vs. control group were − 6.43 (-18.28 to 5.44), -3.98 (-15.95 to 7.98), and − 2.44 (-14.21 to 9.31), respectively.

### Secondary outcomes (Pain and disability)

#### Pain

The study evaluated pain scores over the course of 10 sessions of intervention using a 100 mm VAS. This scale is commonly used to assess the intensity of pain, with 0 mm typically indicating no pain and 100 mm indicating the worst possible pain. The results indicate that there were no statistically significant differences in pain scores between the three groups. Both the main effect of the group (*p* = 0.264) and the group-by-time interaction (*p* = 0.255) were not significant. This means that there were no observable differences in pain scores among the control, compression exercise (CE), and stretching exercise (SE) groups.

**Fig. 6:**
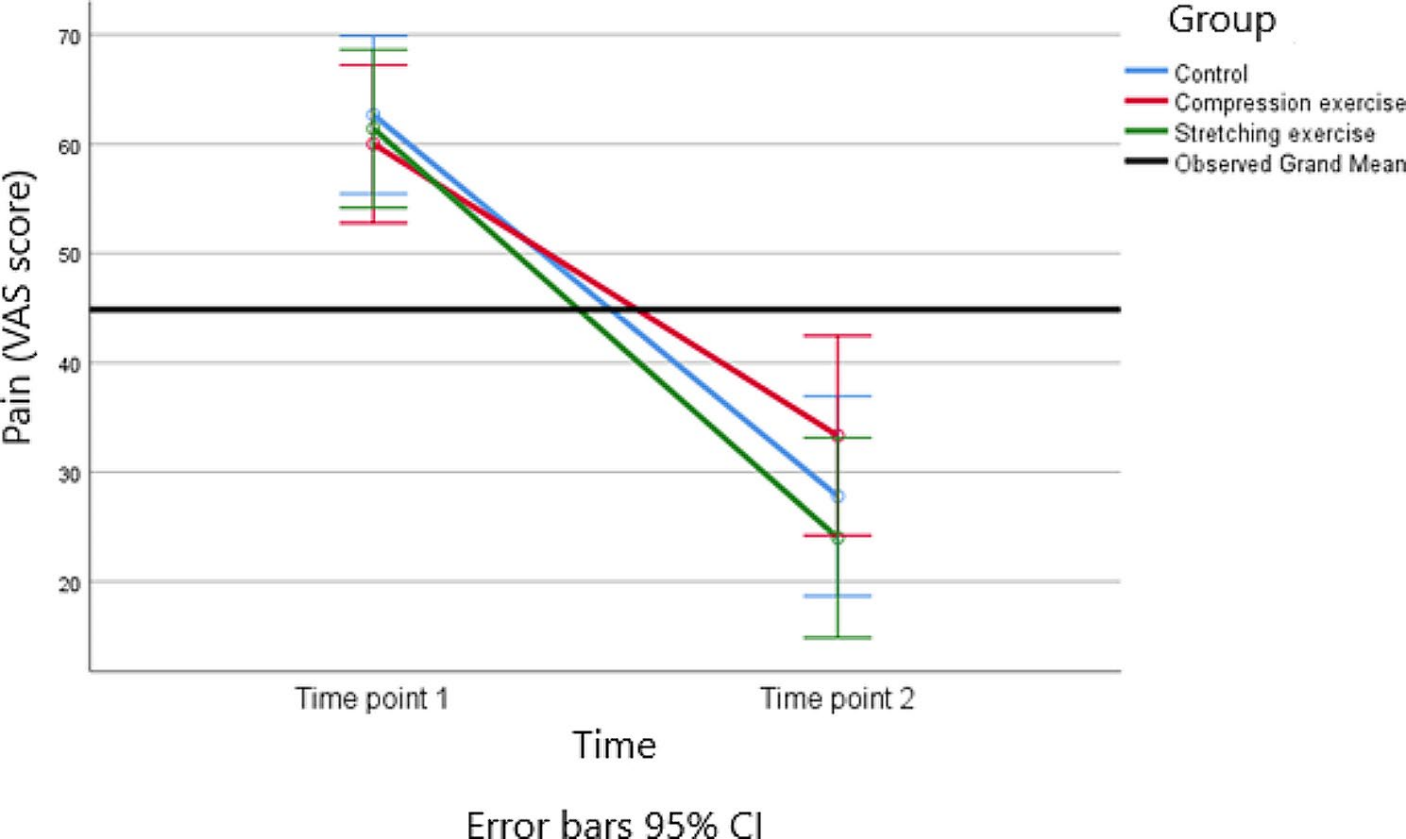
Trends of pain (VAS score) for the three groups at the two time points of the study

However, there was a statistically significant main effect for time (*p* < 0.001). This suggests that pain scores changed significantly over the course of the study. The effect size for this time effect is 0.777, indicating a relatively strong effect. The Fig. 6 illustrates the main effect of time and shows the trends for pain scores across the two study time points (pre vs. post). In all three groups, the scores for pain at the end of the intervention (post-intervention) were significantly reduced when compared to the baseline (pre-intervention). This reduction in pain scores suggests that the interventions had an impact on reducing pain over the course of the study.

#### Disability

**Table 3:** Comparison of pain and disability among the three groups of study (*n* = 45)

Variables	Measurement period	Baseline	After intervention	Main effect of group	Main effect of time	Interaction effect (time*group)
Pain	Compression Exercise	60.00(14.47)	33.33(11.95)	p = 0.264; F = 1.37; df = 2;	p < 0.001; F = 146.22;df = 1; effect size = 0.777	p < 0.255; F = 1.41; df = 2; effect size = 0.063
	Stretching Exercise	61.40(15.1)	24.00(15.49)	effect size = 0.063		
	Control	62.67 (14.37)	27.80(21.75)			
Disability	Compression Exercise	57.16(11.26)	45.80(11.02)	p = 0.009; F = 5.35; df = 2; effect size = 0.207	p = 0.001; F = 85.67; df = 1; effect size = 0.671	p = 0.012; F = 4.89; df = 2; effect size = 0.189
	Stretching Exercise	61.59(15.6)	34.53(8.85)			
	Control	58.75(11.96)	40.20(12.97)			

Mean (SD) and Effect size were reported

The analysis showed that there was a significant difference in disability scores between the three groups (stretching exercise, compression exercise, and a control group). The *p*-value associated with this difference is 0.009, indicating statistical significance. After identifying the significant difference in disability scores among the groups, a post hoc test was conducted to further examine these differences. The post hoc test revealed that the improvement in disability in the stretching exercise group was significantly greater than that in the compression exercise group. The mean difference in disability scores was − 12.62, with a 95% confidence interval ranging from − 20.41 to -4.38, and the *p*-value was again 0.009. The effect size, indicated as 0.207, provides an additional measure of the magnitude of the difference between the two groups. In simpler terms, this means that participants in the stretching exercise group experienced a more significant reduction in their disability scores compared to those in the compression exercise group.

**Fig. 7:**
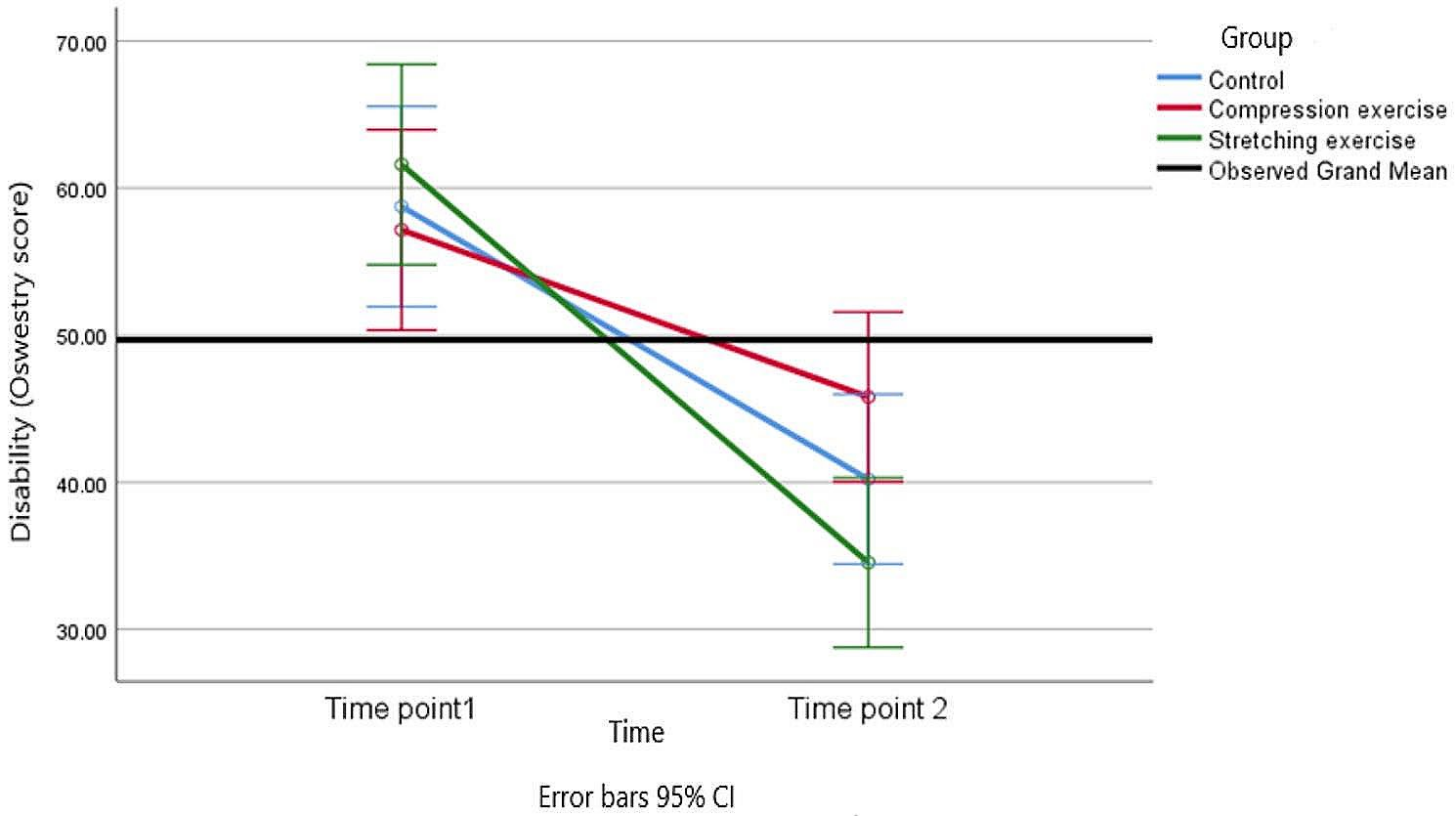
Trends of the disability (Oswestry score) for the three groups at the two time points of the study

The text mentions “Figure 7,” which likely contains a graphical representation of the interaction effect (time*group). This figure visually illustrates the trends in disability scores across the two time points. Among the three groups, it is clear from the figure that disability scores at the end of the intervention (after the exercises) were significantly reduced when compared to the baseline (before the exercises). Furthermore, the figure demonstrates that the stretching exercise group had a significantly larger improvement in disability scores compared to the other two groups (compression exercise and control).

## Discussion

In this study, the primary aim was to compare the effects of stretching and compression exercises on the surface electromyography (sEMG) activity of the gastrocnemius and tibialis anterior muscles in patients with DGS. However, none of the three groups (compression exercise group, stretching exercise group, and control group) demonstrated a statistically significant change in the sEMG activity of the gastrocnemius or tibialis anterior muscles. After the intervention period, there was no statistically significant difference in the change in sEMG activity of the muscles between the three groups. Furthermore, the findings indicated that patients who performed stretching exercises improved in their disability compared to the other groups. Pain and disability decreased significantly in all three study groups after the intervention.

It seems logical that increased pressure on a nerve can indeed affect its function, and this functional impairment could potentially be reflected in electromyography (EMG) signals [12]. To the knowledge of the authors, no study has investigated the effects of DGS treatment on the surface EMG of sciatic nerve muscles. We could not find any related studies on this subject. However, there are many papers on the findings of clinical EMG on this topic in the literature.

In the diagnosis of piriformis syndrome (PS), the absence of a dependable objective test has led to the continued reliance on electromyography (EMG) as the primary method for assessing sciatic nerve impingement [8]. Introduced by Paul Hoffmann in 1918, the H-reflex, characterized by its sensory afferent, synapse, and motor efferent segments, is employed to measure conduction velocity in proximal nerve segments as well as most nerve root segments [13, 14]. The H-reflex is especially informative in cases of potential piriformis muscle compression of the sciatic nerve, particularly when the patient is in the FAIR (flexion, adduction, and internal rotation) position. In such circumstances, if the sciatic nerve experiences compression by the piriformis muscle, it can result in a noticeable delay in the H-reflex.

In their study, Najdi et al. (2019) found that in the diagnosis of piriformis syndrome (PS), a delay in the latency of the peroneal nerve H-reflex is a reliable and effective indicator. They also suggested that in patients exhibiting clinical signs of PS, it may be possible to determine the severity of the affected segment of the sciatic nerve based on threshold values associated with this reflex [8]. Importantly, their research indicated that changes in the latency of the peroneal H-reflex were more dependable as diagnostic markers compared to changes in its amplitude [8].

Another significant finding from this study was the absence of latency change in the posterior tibial H-reflex between the rest and FAIR positions in the affected leg. In contrast, the peroneal H-reflex exhibited a delay under these conditions. The observed delay in the peroneal H-reflex was attributed to the fact that the fibers of the peroneal nerve are located more posteriorly within the sciatic nerve compared to the tibial nerve [8, 15]. This anatomical positioning renders the peroneal nerve more susceptible to compression and results in a greater degree of laxity in the tibial nerve relative to the peroneal nerve [8, 15]. The variation in results between the H-reflex and surface electromyography (sEMG) can be attributed to the distinct methodologies for neuromuscular assessment. While the H-reflex assesses the current compression on the sciatic nerve, sEMG evaluates the overall muscle contraction. These differing assessment methods can yield dissimilar findings, as they provide insights into different aspects of neuromuscular function and responses.

In a study conducted by Nakamura et al., an alternative electrodiagnostic method involving evoked potentials was employed for the diagnosis of PS. In this method, the peroneal nerve was stimulated at the fibular head, and the resulting evoked potential was recorded using an epidural electrode placed at the lumbar spine. The researchers concluded that this evoked potential method is a valuable and useful diagnostic tool for identifying PS [16].

In a study conducted by Chang and Lien [17], they employed a method involving the stimulation of nerve roots using a monopolar needle and the stimulation of the sciatic nerve at the gluteal fold. They measured the motor nerve conduction velocity of the sciatic nerve to assess its condition [17]. While this approach can be utilized to evaluate conditions such as radiculopathy and PS, it’s important to note that electrical stimulation of a nerve with a needle is an invasive and painful procedure [17, 14]. Chein-Wei Chang et al. concluded that the utilization of magnetic nerve stimulation, as a painless, noninvasive, and objective method, enables the assessment of the performance and function of the sciatic nerve in patients with piriformis syndrome [15].

Miller’s perspective suggests that electrodiagnostic tests often yield normal results in patients clinically diagnosed with PS. These tests play a critical role in ruling out more prevalent conditions and assessing the differential diagnosis, which may include conditions like peroneal nerve entrapment, L5 radiculopathy, or sciatic nerve palsy [18]. A controversy exists concerning the utilization of nerve root stimulation and other electrodiagnostic tests such as EMG in the context of DGS assessment.

We suspected the compression on the nerve in the FAIR position may cause contraction disturbance in related muscles and result in decreased muscle activity and normalized RMS. In our study, after a period of stretching and compression exercises, we expected more nerve release and therefore better muscle activity. Nevertheless, there was no change in the normalized RMS of muscles after the exercise interventions. It suggests that this variable might not be sensitive enough to detect changes in muscle activity in the FAIR position due to nerve entrapment. Another possibility is that the sciatic nerve impingement, as experienced in the FAIR position, may not have exerted enough pressure to produce noticeable motor nerve symptoms as measured by EMG. It’s a valuable insight to consider differentiating participants based on the presence or absence of motor symptoms at the beginning of your study. Doing so could have provided a clearer understanding of how your interventions affect the electromyography (EMG) behavior of affected muscles in this syndrome. This approach would allow you to compare the responses of individuals with motor symptoms to those without, shedding light on the effectiveness of your interventions for specific subgroups. Additionally, conducting future studies that focus on comparing RMS in maximal muscle contraction can indeed help clarify this issue. Such studies may provide more precise insights into the impact of interventions on muscle activity and help tailor treatment strategies for individuals with different symptom profiles. This approach could potentially lead to more targeted and effective treatment plans for patients with the syndrome.

We do not know how nerve compression affects the muscle sEMG signal and whether or not the extent of muscle contraction corresponds to sustained compression on the nerve. Studying nerve compression and its precise effect on sEMG could be a new area of research.

The improvement in disability observed in the stretching exercise group compared to the other groups might seem challenging to explain. Rituraj Verma et al.‘s study (2021), which suggests that piriformis stretching is more effective in reducing pain and disability in patients with lumbar disc herniation, provides valuable insights [19]. Their findings indicate that even short-term piriformis stretching can improve the ability to carry out daily activities. It seems more work is needed on this issue [19].

The reduction in pain in the treatment groups is justifiable, as therapeutic interventions and exercises in physiotherapy generally lead to pain management in patients.

### Limitation of study

. Due to the nature of the study and the interventions involved, it was not possible to blind the subjects to their interventions. The randomization process did not consider stratification by sex, leading to an unbalanced distribution of sexes within the study groups. This issue can be considered one of the limitations of our study.

## Conclusion

The authors found that neither stretching nor compression exercises had a significant impact on the sEMG activity of the gastrocnemius and tibialis anterior muscles in patients with DGS. However, stretching exercises did lead to a notable improvement in disability when compared to the other groups. This suggests that stretching exercises may have a positive effect on reducing disability in DGS patients, and further investigation is warranted to explore the potential benefits of these interventions in a more comprehensive manner.

## Data Availability

The data collected and analyzed in the present study are not publicly available due to ethical restrictions but are available from the corresponding author upon request.

## Abbreviations

ANCOVA: Analysis of Covariance
CE: Compression exercise
DGS: Deep gluteal syndrome
EMG: Electromyography
FAIR: Flexion Adduction and Internal Rotation test
LBP: Low back pain
MSDs: Musculoskeletal disorders
PS: Piriformis syndrome
SE: Stretching exercise
TENS: Transcutaneous electrical nerve stimulation
VAS: Visual analogue scale
